# Editorial: Exploration of the Physiological Effects of Exercise in Cardiovascular Diseases

**DOI:** 10.3389/fphys.2020.01097

**Published:** 2020-09-08

**Authors:** Helena Lenasi, Ines Drenjancevic, Markos Klonizakis

**Affiliations:** ^1^Faculty of Medicine, University of Ljubljana, Ljubljana, Slovenia; ^2^Faculty of Medicine, Josip Juraj Strossmayer University of Osijek, Osijek, Croatia; ^3^Scientific Centre of Excellence for Personalized Health Care University Josip Juraj Strossmayer Osijek, Osijek, Croatia; ^4^Lifestyle Exercise and Nutrition Improvement (LENI) Research Group, Sheffield Hallam University, Sheffield, United Kingdom

**Keywords:** exercise, cardiovacsular disease(s), microcirculation, macrocirculation, hemodymamics, exercise physiology

With the cost of treatment for cardiovascular (CV) diseases increasing exponentially every year (Vandenberghe and Albrecht, 2019) it is important to find adjunct therapies to compliment established treatments. These should be sufficiently effective to either reverse or slow-down the progression of these diseases and conditions, not only enhancing treatment, but also improving patients' quality of life. Exercise has been earmarked as one of the main lifestyle components that could be introduced in therapeutic interventions, as it is usually easy to implement by facilitators and be followed by clinical populations (i.e., Klonizakis et al., 2018; Mitropoulos et al., 2020), offering also societal and quality of life benefits (Kesterton et al., 2019).

Nevertheless, exploring the physiological effects of exercise-based interventions is commonly neglected, with the main focus of studies being given to the interventions' therapeutic contribution. In addition, there is limited knowledge on the methods to either diagnose patients with some borderline diseases (or atypical symptoms) or to trace the efficiency of therapeutic approaches (in patients); to this end, new methods are emerging, helping to detect patients at risk or the response to exercise.

This Research Topic brings together contributions from researchers to advance our understanding as of how exercise affects the vascular physiology of clinical populations, allowing us to take valuable lessons and transfer the gained knowledge further.

Fanget et al. showed that establishing the force-velocity-power (FVP) relationship can support the assessment of the dynamic force production capacities in coronary artery disease (CAD) patients. In their study, mechanical parameters (e.g., theoretical maximum force, velocity, and the maximal power output) were determined during cycloergometer sprint sessions to estimate the FVP relationship slope. They suggest that the observed differences between patients and healthy individuals reflect loss of muscle mass, remodeling of motor units or a neuromuscular activation deficit, and potentially be of value for training adjustment and optimization.

A more invasive approach was applied by Chen et al. in a cohort of heart failure patients with preserved ejection fraction (HFpEF). This clinical group can be difficult to diagnose, as many patients remain asymptomatic at rest, but develop symptoms during exercise. Thus, it would be desirable to predict the response during exercise by using non-invasive measures. The authors showed that the stress echocardiography-derived E/e' ratio (e.g., *the ratio of early mitral inflow velocity to early diastolic tissue velocity*), which is a measure of diastolic function, can be a reliable predictor of abnormal exercise hemodynamics in HFpEF patients.

Chronic obstructive disease (COPD) patients are another high-risk group, for whom the assessment of respiratory muscles function (at rest and during exercise) can yield useful information. Electromyography (EMG) of the diaphragm, coupled to simultaneous cardiac activity tracing by electrocardiogram (ECG) might represent an indirect measure of neural respiratory drive. As ECG tracings are prone to artifacts Dacha et al. developed a semi-automated protocol for ECG artifacts removal during transesophageal diaphragm EMG. Their findings suggest that the proposed semi-automated method can reliably be used to evaluate changes in EMG amplitudes over a wide range of minute ventilation recorded at rest and during exercise testing in COPD patients. In addition, compared to manual methods, the presented method is more time-efficient, and exhibits less inter-rater variability, and as such could be regarded as a reliable new standard for objective EMG amplitude analyses in future clinical and research settings.

Henni et al. have shown that transcutaneous oxymetry (TcPO_2_) is a feasible and reliable, non-invasive clinical test to assess microvascular responsiveness in patients with thoracic outlet syndrome (TOS), regardless of the underlying etiology. Using the standardized Roos test, applied to challenge microcirculation, they showed that the TcPO_2_ drop was correlated either with the clinical symptoms or with the ultrasonographic results allowing the detection of TOS in patients. In addition, TcPO_2_ can be applied to track potential benefits of surgical or conservative (e.g., kinesotherapy) therapies.

Stupin et al. elucidated the benefits of n-3 polyunsaturated fatty acids (PUFAs) which have been confirmed in several studies as an effective dietary supplement to induce pleiotropic physiological effects, on CV, muscular and immune system, in healthy and in CV patients. Potential mechanisms of PUFAs action on CV system are presented, from their antithrombotic and anti-inflammatory effects, to their potential to improve endothelial (dys)function, respectively reducing the risk of CV events'. Moreover, PUFA's have also been suggested to modulate oxygen consumption during intense exercise which may be beneficial in increasing metabolic capacity, shifting the anaerobic threshold, and accordingly diminish the delayed-onset muscle soreness. Yet, the results of various studies are inconclusive with respect of the effects of PUFAs in men and women, in healthy and patients, and regarding their interaction with exercise. To this end, the paper might encourage researchers to perform additional mechanistic, epidemiologic and clinical studies on PUFAs.

The study by Vasić et al. represents a starting point for further research into optimal exercise modalities in CAD with a recent myocardial infarction or revascularization procedure, with water-based training likely emerging as a suitable exercise option. Endurance plus calisthenics exercise training in thermo-neutral water has been shown as safe, improving aerobic exercise capacity and vascular function in patients undergoing short-term residential cardiac rehabilitation after a recent CAD event.

A review paper by Guo et al. discusses the cardioprotective factors of exercise after myocardial infarction (MI). These factors are secreted by or enriched in the heart and execute their action in an autocrine or paracrine manner. The paper focuses on Growth Differentiation Factor 15 (GDF15), Exercise-induced Follistatin-Like1 (FSTL1), Non-coding RNAs (ncRNAs), Cardiac-derived miRNAs, longcRNAs and Cardiomyocytes secrete extracellular vesicles. All of these may be novel targets to study the mechanism of exercise-induced benefits, besides traditional signaling pathways.

Physical activity is an efficient strategy to delay development of obesity and insulin resistance, and thus the progression of obesity/diabetes-related cardiomyopathy. The study by Boardman et al. explored the effect of exercise on ischemic-tolerance when exercise was initiated after the development of obesity-mediated cardiomiopathy in high-fat fed mice.

The authors present the beneficial effects of exercise training with regard to improving the ischemic-tolerance in hearts with cardiomyopathy following obesity and insulin resistance. This study also emphasizes the exercise-induced improvement of cardiac energetics and mitochondrial function in obesity/diabetes.

Finally, Kambič et al. presented the results of assessment of the safety and efficacy of blood-flow restricted (BFR) resistance training in patients with stable CAD compared to usual care. Eight weeks of BFR resistance training did not show any unfavorable cardiovascular responses (such as hemodynamic alterations, anginal symptoms, or ventricular arrhythmias) but was associated with significant improvements in muscle strength and decrease in systolic blood pressure. Thus, it may be therefore provided as an additional exercise option to aerobic exercise to improve skeletal muscle functioning in patients with CAD.

In conclusion, the topic encompasses a heterogenous range of papers, that are relevant from the clinical point of view as they can broaden therapeutic strategies and challenge researchers to further study and elucidate unsolved questions. We look forward that this knowledge will be used in further, larger trials and translated to sustained clinical practice.

## Author Contributions

All authors contributed equally to the first draft of the manuscript, manuscript revision, and read and approved the submitted version. HL and ID share first authorship of this publication.

## Conflict of Interest

The authors declare that the research was conducted in the absence of any commercial or financial relationships that could be construed as a potential conflict of interest.
